# Results from an LGBTQ+ Community Health Needs Assessment in Nassau and Suffolk Counties of New York State

**DOI:** 10.1007/s10597-022-01069-8

**Published:** 2023-02-11

**Authors:** Allison H. Eliscu, Jennifer Jamilkowski, Adam Gonzalez, Jennifer Mesiano Higham, Lucy Kenny, Margaret M. McGovern

**Affiliations:** 1 Department of Pediatrics, Renaissance School of Medicine at Stony Brook University, HSC 11-060, Stony Brook, NY 11794 USA; 2 Department of Planning, Stony Brook University, Stony Brook, NY USA; 3 Department of Psychiatry and Behavioral Health, Renaissance School of Medicine at Stony Brook University, Stony Brook, NY USA; 4 Yale School of Medicine, Yale University, New Haven, CT USA

**Keywords:** Community Needs Assessment, Health Disparities, LGBTQ+, Sexual and Gender Minority, Access to Care, Health-Related Behaviors

## Abstract

LGBTQ+ individuals experience health care disparities and difficulty accessing affirming care. Little is known regarding the health and experiences among subpopulations of specific sexual orientations and gender identities (SOGI). We implemented the first LGBTQ + health needs assessment survey in Nassau and Suffolk Counties, New York, to assess individuals’ health care experiences, behaviors, access to care, and health care needs. The sample (N = 1150) consisted of many SOGI subgroups. Greater than 60% of respondents reported symptoms of chronic depression; over one third reported disrespectful health care experiences; and two thirds experienced verbal harassment. Bisexual/bicurious, pansexual, queer, gender nonconforming and transgender individuals experienced highest rates of mental health concerns and difficulty accessing care. Behavioral health concerns were also high among Black, multiracial, Hispanic, Asian, young adult, and lower-income respondents. Gaining an understanding of unique differences among LGBTQ+ subgroups can guide implementation of services targeting specific subpopulations to improve access to care and reduce disparities.

## Introduction

Lesbian, gay, bisexual, transgender, queer and other sexual and gender diverse individuals (LGBTQ+) experience significant health disparities (Institute of Medicine, 2011). Compared to heterosexual and gender binary peers, LGBTQ+ individuals are 2.5 times more likely to experience anxiety, depression and substance misuse (Cochran et al., 2003). This population also experiences higher rates of suicidal ideations and attempts (Herman et al., 2019; Massachusetts Department of Health 2009; Semlyen et al., 2016); negative health behaviors (e.g., alcohol and tobacco use, physical inactivity, obesity) (Buchting et al., 2017; Centers for Disease Control and Prevention 2022b; Division of Diversity and Health Equity, 2017; Gay and Lesbian Medical Association 2001; Medina et al., 2021); sexually transmitted infections (Centers for Disease Control and Prevention 2016; Shover et al., 2018); and physical health conditions (Abd-Elsayed et al., 2021, Fredriksen-Goldsen et al., 2017). In 2016, the National Institutes of Health officially designated LGBTQ+ individuals as a health disparity population for research (Perez-Stable, 2016). Since then, many medical organizations have created guidelines emphasizing the need for affirmative and inclusive care (American Academy of Family Physicians, 2020; American College of Obstetricians and Gynecologists, 2021; American Nurses Association Center for Ethics and Human Rights, 2018; Council on Minority Mental Health and Health Disparities, 2020; Daniel et al., 2015; Rafferty et al., 2018). Goals to reduce health disparities for the LGBTQ+ population are also highlighted in the national Healthy People 2030 initiative (Office of Disease Prevention and Health Promotion. (n.d.) Healthy People 2030: LGBT https://health.gov/healthypeople/objectivesanddata/browse-objectives/lgbt).

LGBTQ+ individuals are less likely to receive preventive health services. For example, identifying as gender non-conforming, transgender or bisexual is associated with a lower likelihood of obtaining routine breast, cervical and colon cancer screenings (Bazzi et al., 2015; Johnson et al., 2016; Tabaac et al., 2018). Research has identified many contributing factors which may deter LGBTQ+ individuals from receiving medical care (Haviland et al., 2020). Studies have shown between one third to one half of transgender individuals have experienced a negative interaction with a health care provider (Bradford et al., 2013; Jaffee et al., 2016; James et al., 2016; Medina et al., 2021; Shires & Jaffee, 2015). Many LGBTQ+ individuals also feel providers are not educated and experienced to care for their needs (Qureshi et al., 2018). In one study, transgender and gender nonconforming individuals were four times more likely to delay care if they felt the need to educate their providers about appropriate care (Jaffee et al., 2016). In fact, Healthy People 2020 acknowledged a shortage of knowledgeable and culturally competent providers for LGBTQ+ care (Office of Disease Prevention and Health Promotion, 2014). While a modest body of literature documents the health disparities in the LGBTQ+ population, needs vary by demographic factors (McCann & Brown, 2019; Stepleman et al., 2019) and subgroups of this population also vary significantly from one another. Research is often limited by combining LGBTQ+ individuals into one or limited subgroups with few studies actually considering the unique experiences of diverse sexual orientations and gender identities (SOGI) (Feinstein et al., 2020; Fiani & Han, 2018; Hutsell, 2012; Matsuno & Budge, 2017; Smalley et al., 2016). It is essential to gain a greater understanding of the health care needs of these historically marginalized, stigmatized, and understudied populations in order to improve health outcomes.

The goal of this study was to conduct a community health needs assessment among LGBTQ+ adults residing or enrolled in an educational program in Nassau and Suffolk Counties in New York State (NYS). Data will be used to inform the development of LGBTQ+ health equity initiatives that improve the LGBTQ+ community health status in Nassau/Suffolk Counties; reduce LGBTQ+ health disparities; increase accessibility to preventative health services; and improve provider training.

## Methods

A community health needs assessment was conducted with close collaboration by Professional Research Consultants, Inc. (PRC) and over 30 community partners, including health care providers, social services providers, advocacy and public health organizations, county government officials, university students and administrators, informal community leaders, artists, entertainers and bar/restaurant owners. The community partners were critical to this study; providing their perspective on issues facing the local community, recruiting focus group participants, and promoting the survey to constituents.

### Study Design

This study was exploratory and cross-sectional, consisting of an anonymous, online survey among a convenience (snowball) sample of adults (ages 18+) identifying as LGBTQ+ and residing or enrolled in an educational program in Nassau/Suffolk Counties. A convenience sample was used as there is no data available on the size of the region’s LGBTQ+ population, and some individuals are not comfortable disclosing their LGBTQ+ identity. Similar recruitment methods have been used for other LGBTQ+ health needs assessments (Coleman et al., 2014; Stepleman et al., 2019).

The survey was offered in both English and Spanish from June through September 2021 and took approximately 15–20 min to complete. Respondents had the option of skipping questions they found triggering. Preceding each potentially triggering section was a brief description of that section’s subject matter, links to local and national support resources, and an option to forgo that section. No respondent identifiers were retained, including IP addresses. The survey was approved by the university's Institutional Review Board.

### Survey Design

Several validated and widely used surveys were used as a starting point for this survey instrument including the Centers for Disease Control and Prevention (CDC) Behavioral Risk Factor Surveillance System (BRFSS) (29 questions) (Centers for Disease Control and Prevention, 2022a); PRC community health needs assessments (24 questions); and the Patient Health Questionnaire-4 (PHQ-4) which is a validated brief screening tool to detect anxiety and depression disorders (4 questions) (Kroenke et al., 2009). The majority of questions (90 questions) and survey domains were developed based on input from key stakeholder meetings and focus groups with diverse LGBTQ+ individuals, held in English and Spanish. The survey was also inspired by national (James et al., 2016) and international surveys (European Union Agency for Fundamental Rights, 2012) especially for survey domain selection, survey promotion, and strategies to reach the target population.

Survey questions focused on the following topics: access to care; transgender health needs; experiences in health care and the community at large; health status and health-related behaviors; mental health needs; experiences with harassment and violence; and LGBTQ+ priority issues. Demographic information collected included: age, race, ethnicity, SOGI, transgender identity and household income. All respondents were asked if they identify as transgender regardless of their gender identity response. Response fields for SOGI offered auto complete options (7 auto-response options for sexual orientation and 26 for gender identity), but respondents could enter any response into these fields. Combining a type assist feature for entering common gender and sexual identity terms, while still providing respondents the ability to free-text their sexual orientation and gender identity reduced the number of typographic errors and gave the community full flexibility to self-identify. The ability to flexibly self-identify (versus forced choice options) was noted as an important survey feature for LGBTQ+ individuals by focus group members and collaborative partners. When possible, the survey questions were taken from validated screening tools (Centers for Disease Control and Prevention, 2022a) to facilitate the comparison of findings to the general adult population in Suffolk County (Suffolk), NYS, and across the US, allowing the study team to understand the extent of disproportionate health care needs or disparities within the region’s LGBTQ+ population.

### Survey Distribution

During the survey period, URL and QR codes for the survey were distributed throughout Nassau/Suffolk Counties via electronic and in-person promotions. Electronic promotions included social media posts, a web-based panel discussion, television and online dating application advertisements, and email blasts. In-person promotion occurred at regional Pride events, LGBTQ+ friendly venues, and at community partners’ events. PRC and the study team monitored response rates to understand whether any respondent subgroups based on geographic or demographic factors were underrepresented. When any group was poorly represented, promotional strategies were deployed, and new partnerships were developed targeting those communities.

### Data Analysis

Surveys exhibiting inconsistent or implausible response patterns were removed (n = 68). Data analysis included a summary of the raw data and comparison of responses based on demographic and SOGI groups. Regarding sexual orientation, identities with less than 50 responses were combined into a category entitled “Identities < 50”. Regarding gender identity, identities with less than 50 responses were combined with “Nonbinary” into a category entitled “Gender Nonconforming”.

Survey data were compared to Suffolk, NYS and US data where questions aligned, using benchmark data from the 2018 Suffolk County Community Health Needs Assessment (PRC Inc, 2018), CDC BRFSS (NYS comparisons), and the 2020 PRC National Health Survey (US comparisons) (PRC Inc, 2021). Benchmark data were not available for Nassau County. However, understanding that the convenience sampling methodology yields a sample that is less representative than a true random sample, it was important to adjust the LGBTQ+ sample responses to minimize the effect of disparities resulting from sampling differences. Thus, survey data compared to county, state, and national data were adjusted to match overall county-level demographics for key demographic characteristics (namely sex, age, race, and ethnicity). To achieve this, SPSS applied weighting variables that produced an adjusted sample, which more closely matches the general population for these characteristics; the assumption here is that the true LGBTQ+ population is roughly proportionally equal within each of these demographic cohorts. With this weighting practice, while the integrity of each individual’s responses is maintained, one respondent’s answers may contribute to the whole more than others; another respondent, whose demographic characteristics may have been oversampled, may contribute less than others.

## Results

1,150 validated surveys were returned. Demographic data is represented in Table 1. The numbers and percentages reported in Table 1 represent the total number of respondents and percentage of all respondents identified in each subcategory. The mean age of respondents was 36.1 (range 18–86 years). More than half (53.8%) reported a mid/high household income. Over 1 in 5 (21.6%) were categorized as very low-income earning < 200% of the federal poverty level. Most identified as White (78.1%), and 20.1% as Hispanic. Respondents provided diverse answers when asked to describe their SOGI. There were 25 unique responses for sexual orientation which, for statistical purposes, were grouped into gay man (28.8%), lesbian (24.8%), bisexual/bicurious (22.4%), pansexual (8.0%), queer (8.8%), and other (“Identities < 50”) (6.0%). Regarding gender identity, there were 34 unique responses which were grouped into identifying as male (36.8%), female (47.9%) and gender nonconforming (15.1%). Overall, 13.2% of all respondents identified as transgender; 33.8% of transgender respondents identified their gender identity as male, 23.0% as female and 43.2% as gender nonconforming. Tables 2, 3, 4, 5 and 6 detail responses by sexual orientations, gender identities and other demographic factors for mental health, health-related behaviors, sexual health, access to care, and harassment and violence outcomes. Data in these tables are organized horizontally by demographic category and subcategory. Each cell contains the number of people who responded affirmatively to that question. The percentages reported beside each value in parentheses indicate the percentage of respondents in that given demographic subcategory who responded to the particular question being highlighted in the table. Below, overall sample rates and comparisons with the general Suffolk County, NYS and US populations are provided, where possible.

**Table 1:** Demographics

Demographic category	Subcategory	N = 1150 (%)
Gender Identity	Identifies Male	421 (36.9%)
	Identifies Female	548 (48.0%)
	Gender Nonconforming^a^	172 (15.1%)
Transgender Status	Transgender	152 (13.2%)
	Not Transgender	996 (86.8%)
Sexual Orientation	Gay Man	330 (29.1%)
	Lesbian	285 (25.1%)
	Bisexual/bicurious	257 (22.7%)
	Pansexual	92 (8.1%)
	Queer	101 (8.9%)
	Identities < 50^b^	69 (6.1%)
Age Bracket	18–25 years old	389 (33.8%)
	26–39 years old	378 (32.9%)
	40–64 years old	308 (26.8%)
	65 + years old	75 (6.5%)
Student status	Student	382 (33.2%)
	Non-student	768 (66.8%)
Race	White	890 (78.1%)
	Asian	58 (5.1%)
	Black/African American	47 (4.1%)
	Multiracial	139 (12.2%)
	Other	7 (0.6%)
Ethnicity	Hispanic (Any Race)	231 (20.1%)
Income Bracket^c^	Very Low-Income	239 (21.6%)
	Low-Income	272 (24.6%)
	Mid/High-Income	595 (53.8%)

^a^“Gender Nonconforming” combines nonbinary (8.0%) with gender identity responses with less than 50 respondents including: Gender Fluid 1.8%, Gender Queer 1.8%, Queer 0.5%, Gender Questioning 0.4%, Agender 0.4%, Bigender 0.3%, Androgyne/Androgynous 0.3%, Crossdresser 0.2%, Bisexual 0.2%, Gender Nonconforming 0.2%, Questioning 0.2%. Additional responses, each comprising 0.1% of identities, are: Neither, Asexual, Transgender Nonbinary, Pansexual, Neutrois, Intergender, Transgender, Pangender, Alien

^b^Sexual orientation responses with less than 50 respondents were combined into the category “Identities < 50” including: Asexual 2.6%, Heterosexual 2.2%, Questioning 0.9%. Additional responses, each comprising 0.1% of identities, are: Polysexual, Cupiosexual, Homoromantic Asexual, Aromantic Grey – Asexual

^c^“Very low-income”, “low-income” and “mid/high-income” refers to individuals living in a household earning < 200%, 200–399% and > 400% of the federal poverty level respectively (based on The US Departments of Health & Human Services administrative poverty thresholds defined by household income level and number of persons in the household)

### Mental Health

Respondents reported significant mental health difficulties (Table 2). Nearly half of the sample (43.6%) rated their mental health as “fair” or “poor.” More than a third (37.5%) screened positive for moderate to severe anxiety/depression. Nearly two-thirds (61.5%) reported symptoms of chronic depression. In the past three years, 33.5% of all respondents had thoughts of self-harm, 16.8% had intentionally self-injured, 23.9% seriously considered suicide and 3.7% attempted suicide.

**Table 2:** Mental health

Demographic Category	Subcategory	Overall mental health fair/poor	Moderate to severe anxiety or depression^a^	Chronic depression^b^	Self-harm thoughts in past 3 years	Self-harm in past 3 years	Seriously considered suicide in past 3 years	Suicide attempt in past 3 years	Currently receiving mental health treatment
Gender Identity	Male	129 (30.6%)	116 (27.7%)	221 (52.5%)	76 (20.5%)	36 (9.7%)	59 (15.9%)	5 (1.4%)	112 (26.6%)
	Female	246 (45.0%)	201 (36.9%)	337 (61.6%)	169 (34.6%)	79 (16.2%)	109 (22.4%)	17 (3.5%)	213 (38.9%)
	Gender Nonconforming^c^	122 (70.6%)	107 (62.2%)	144 (83.2%)	93 (59.6%)	54 (34.6%)	73 (46.8%)	14 (9.0%)	77 (44.5%)
Transgender Status	Transgender	101 (66.9%)	80 (53.0%)	128 (84.8%)	81 (59.6%)	48 (35.0%)	61 (44.9%)	9 (6.6%)	72 (47.7%)
	Not Transgender	399 (40.1%)	348 (35.1%)	578 (58.0%)	260 (29.4%)	124 (14%)	183 (20.7%)	29 (3.3%)	331 (33.2%)
Sexual Orientation	Gay	86 (26.1%)	80 (24.5%)	159 (48.2%)	47 (16.0%)	15 (5.1%)	46 (15.7%)	7 (2.4%)	85 (25.8%)
	Lesbian	99 (34.8%)	89 (31.4%)	154 (54.0%)	65 (25.9%)	41 (16.3%)	42 (16.7%)	4 (1.6%)	110 (38.7%)
	Bisexual/ Bicurious	139 (54.3%)	112 (43.7%)	180 (70.3%)	96 (42.3%)	52 (22.9%)	67 (29.6%)	9 (4.0%)	98 (38.1%)
	Pansexual	64 (69.6%)	55 (59.8%)	72 (78.3%)	51 (62.2%)	25 (30.5%)	30 (36.6%)	8 (9.8%)	41 (44.6%)
	Queer	66 (65.4%)	53 (52.5%)	77 (76.2%)	55 (59.8%)	26 (28.3%)	37 (40.2%)	5 (5.4%)	40 (39.6%)
	Identities < 50^d^	40 (57.9%)	33 (48.5%)	54 (78.3%)	22 (34.9%)	11 (17.5%)	19 (30.2%)	4 (6.3%)	24 (34.8%)
Age Bracket	18–25 years old	258 (66.5%)	204 (52.6%)	286 (73.7%)	189 (54.0%)	114 (32.4%)	127 (36.3%)	27 (7.7%)	154 (39.6%)
	26–39 years old	169 (44.7%)	148 (39.5%)	249 (65.9%)	113 (34.0%)	48 (14.5%)	78 (23.5%)	10 (3.0%)	112 (32.3%)
	40–64 years old	63 (20.4%)	67 (21.9%)	143 (46.4%)	34 (12.5%)	9 (3.3%)	32 (11.8%)	1 (0.4%)	103 (33.6%)
	65 + years old	11 (14.7%)	10 (13.3%)	29 (38.7%)	6 (8.8%)	1 (1.5%)	7 (10.4%)	0 (0.0%)	25 (33.3%)
Race	White	376 (42.2%)	325 (36.7%)	538 (60.4%)	282 (31.7%)	136 (17.1%)	180 (22.7%)	29 (3.7%)	334 (37.6%)
	Asian	29 (50.0%)	25 (43.1%)	37 (63.8%)	30 (51.0%)	16 (31.4%)	16 (31.4%)	4 (7.8%)	13 (22.4%)
	Black/African American	25 (53.2%)	22 (47.8%)	32 (68.0%)	20 (43.6%)	6 (15.4%)	14 (35.9%)	1 (2.6%)	17 (36.2%)
	Multiracial	58 (46.7%)	53 (38.1%)	91 (65.5%)	49 (35.2%)	14 (11.4%)	33 (26.8%)	4 (3.3%)	36 (25.9%)
Ethnicity	Hispanic (Any Race)	96 (41.5%)	80 (34.6%)	148 (64.1%)	69 (29.7%)	29 (13.7%)	46 (22.0%)	8 (3.8%)	58 (25.1%)
Income Bracket^e^	Very Low-Income	140 (58.5%)	116 (48.5%)	171 (71.5%)	92 (44.9%)	45 (22.0%)	69 (33.8%)	14 (6.9%)	81 (33.9%)
	Low-Income	152(55.9%)	126 (46.5%)	187 (68.8%)	97 (38.3%)	52 (20.6%)	69 (27.4%)	6 (2.4%)	94 (34.6%)
	Mid/High-Income	192 (32.3%)	172 (29.1%)	319 (53.6%)	138 (26.3%)	64 (12.1%)	96 (18.3%)	15 (2.9%)	215 (36.2%)
	Total N (%)	501 (43.6%)	429 (37.5%)	707 (61.5%)	342 (33.5%)	172 (16.8%)	244 (23.9%)	38 (3.7%)	404 (35.2%)

^a^Moderate to severe anxiety or depression is based on Patient Health Questionnaire-4 (PHQ-4) scale which assesses for presence of anxiety or depression symptoms during the prior two weeks using a 4 question, 4-point Likert scale (Kroenke 2009). Rates of moderate to severe anxiety or depression were calculated based on the cumulative PHQ-4 score with scores of 6–8 and 9–12 indicating moderate and severe anxiety and/or depression

^b^Chronic depression defined as 2 or more years in their lives when they felt depressed or sad on most days

^c^“Gender Nonconforming” combines nonbinary (8.0%) with gender identity responses with less than 50 respondents including: Gender Fluid 1.8%, Gender Queer 1.8%, Queer 0.5%, Gender Questioning 0.4%, Agender 0.4%, Bigender 0.3%, Androgyne/Androgynous 0.3%, Crossdresser 0.2%, Bisexual 0.2%, Gender Nonconforming 0.2%, Questioning 0.2%. Additional responses, each comprising 0.1% of identities, are: Neither, Asexual, Transgender Nonbinary, Pansexual, Neutrois, Intergender, Transgender, Pangender, Alien

^d^Sexual orientation responses with less than 50 respondents were combined into the category “Identities < 50” including: Asexual 2.6%, Heterosexual 2.2%, Questioning 0.9%. Additional responses, each comprising 0.1% of identities, are: Polysexual, Cupiosexual, Homoromantic Asexual, Aromantic Grey – Asexual

^e^“Very low-income”, “low-income” and “mid/high-income” refers to individuals living in a household earning < 200%, 200–399% and > 400% of the federal poverty level respectively (based on The US Departments of Health & Human Services administrative poverty thresholds defined by household income level and number of persons in the household)

Despite high rates of mental health difficulties, only 35.2% of respondents were currently receiving treatment for a mental health condition. Of those who identified their mental health as “fair” or “poor”, only 46.4% were currently receiving mental health treatment. Nearly half of those who reported self-harm (47.4%) and those who had seriously considered suicide (47.3%) within the prior three years were not currently seeing a mental health provider. Furthermore, nearly half of all respondents with a prior suicide attempt did not receive any follow-up care or support after the episode (47.7%) due to feeling too depressed (25.6%), concern about confidentiality (23.2%), not knowing where to follow-up (22.0%), and lack of insurance (9.8%).

When adjusted to account for demographic differences, survey respondents were more than twice as likely to experience “fair” or “poor” mental health (31.3% adjusted) compared to Suffolk (14.8%) and US (13.4%); and nearly twice as likely to experience symptoms of chronic depression (53.0% adjusted) compared to Suffolk (28.2%) and US (30.3%). Survey respondents (34.0% adjusted) were twice as likely to be receiving mental health treatment compared to US rates (16.8%).

### Health-Related Behaviors

Overall, 28.2% of respondents were obese (BMI ≥ 30). A total of 12.2% of respondents currently smoke cigarettes every day or some days. Over one-third (34.9%) reported excessive alcohol use and 14.6% reported using an illicit drug in the past month (Table 3).

**Table 3:** Health related behaviors

Demographic category	Subcategory	Obesity (BMI > 30)	Current Smoker^a^	Excessive Alcohol Use (past month)^b^	Illicit Drug use (past month)
Gender Identity	Male	97 (23.3%)	196 (17.1%)	472 (41.1%)	186 (16.2%)
	Female	165 (30.6%)	107 (9.3%)	374 (32.6%)	149 (13.0%)
	Gender Nonconforming^c^	56 (32.7%)	106 (9.2%)	318 (27.7%)	179 (15.6%)
Transgender Status	Transgender	42 (28.2%)	137 (11.9%)	334 (29.1%)	212 (18.5%)
	Not Transgender	278 (28.3%)	140 (12.2%)	411 (35.8%)	161 (14.0%)
Sexual Orientation	Gay	68 (20.9%)	209 (18.2%)	483 (42.1%)	196 (17.1%)
	Lesbian	91 (32.6%)	98 (8.5%)	335 (29.2%)	117 (10.2%)
	Bisexual/ Bicurious	67 (26.2%)	152 (13.2%)	449 (39.1%)	197 (17.2%)
	Pansexual	38 (42.7%)	113 (9.8%)	336 (29.3%)	200 (17.4%)
	Queer	32 (31.7%)	46 (4.0%)	352 (30.7%)	171 (14.9%)
	Identities < 50^d^	18 (26.9%)	116 (10.1%)	282 (24.6%)	100 (8.7%)
Age Bracket	18–25 years old	74 (19.2%)	21 (5.4%)	125 (32.1%)	51 (13.1%)
	26–39 years old	121 (32.6%)	57 (15.1%)	169 (44.7%)	71 (18.8%)
	40–64 years old	108 (35.8%)	57 (18.5%)	84 (27.4%)	40 (13.1%)
	65 + years old	17 (23.0%)	5 (6.8%)	23 (31.1%)	5 (6.7%)
Race	White	258 (29.4%)	96 (10.8%)	304 (34.2%)	112 (12.6%)
	Asian	6 (10.5%)	3 (5.1%)	13 (22.4%)	6 (10.3%)
	Black/African American	15 (33.3%)	7 (14.9%)	18 (38.3%)	9 (19.1%)
	Multiracial	37 (27.0%)	32 (23.0%)	62 (44.6%)	37 (26.6%)
Ethnicity	Hispanic (Any Race)	63 (27.6%)	56 (24.3%)	95 (41.3%)	40 (29.0%)
Income Bracket^e^	Very Low-Income	52 (22.1%)	43 (18.0%)	88 (37.0%)	56 (23.7%)
	Low-Income	66 (24.4%)	42 (15.5%)	91 (33.5%)	52 (19.1%)
	Mid/High-Income	193 (33.0%)	54 (9.1%)	221 (37.2%)	57 (9.6%)
	Total N (%)	321 (28.2%)	140 (12.2%)	401 (34.9%)	167 (14.6%)

^a^Currently smoke cigarettes either regularly (daily) or occasionally (on some days)

^b^Excessive alcohol use reflects the number of persons who drank more than two drinks per day on average (for those identifying as male or nonbinary) or more than one drink per day on average (for those identifying as female) OR who drank 5 or more drinks during a single occasion (for those identifying as male or nonbinary) or 4 or more drinks during a single occasion (for those identifying as female) during the past 30 days

^c^“Gender Nonconforming” combines nonbinary (8.0%) with gender identity responses with less than 50 respondents including: Gender Fluid 1.8%, Gender Queer 1.8%, Queer 0.5%, Gender Questioning 0.4%, Agender 0.4%, Bigender 0.3%, Androgyne/Androgynous 0.3%, Crossdresser 0.2%, Bisexual 0.2%, Gender Nonconforming 0.2%, Questioning 0.2%. Additional responses, each comprising 0.1% of identities, are: Neither, Asexual, Transgender Nonbinary, Pansexual, Neutrois, Intergender, Transgender, Pangender, Alien

^d^Sexual orientation responses with less than 50 respondents were combined into the category “Identities < 50” including: Asexual 2.6%, Heterosexual 2.2%, Questioning 0.9%. Additional responses, each comprising 0.1% of identities, are: Polysexual, Cupiosexual, Homoromantic Asexual, Aromantic Grey—Asexual

^e^“Very low-income”, “low-income” and “mid/high-income” refers to individuals living in a household earning < 200%, 200–399% and > 400% of the federal poverty level respectively (based on The US Departments of Health & Human Services administrative poverty thresholds defined by household income level and number of persons in the household)

When adjusted to account for demographic differences, survey data demonstrated disparities compared to Suffolk, state and US data for multiple measures of substance use including current smoking (13.2% of respondents; 10.0% Suffolk, 12.7% NYS, and 17.4% US); excessive drinking (31.7% respondents; 26.9% Suffolk, 18.2% NYS, and 27.2% US); and illicit drug use in past month (12.6% respondents, which is nearly double the rate in Suffolk (6.6%) and six times the national rate (2.0%)).

### Sexual Health

Overall, 60.5% reported ever being tested for HIV and 5.1% of respondents reported testing positive for HIV (Table 4). Additionally, 25.7% of those never tested stated they want to get tested. At the time of the survey, 77.0% of respondents were aware of pre-exposure prophylaxis (PrEP) for HIV prevention and 4.5% of the total sample reported currently using PrEP.

**Table 4:** Sexual health

Demographic Category	Subcategory	Ever Tested for HIV	Positive HIV Status	Aware of HIV PrEP	Current Use of HIV PrEP	History of Sex Work
Gender Identity	Male	298 (74.5%)	49 (12.3%)	303 (86.3%)	37 (10.6%)	28 (6.7%)
	Female	248 (51.1%)	2 (0.4%)	341 (70.7%)	3 (0.6%)	36 (6.5%)
	Gender Nonconforming^a^	77 (52.4%)	2 (1.4%)	115 (74.5%)	4 (2.8%)	28 (16.1%)
Transgender Status	Transgender	73 (53.7%)	1 (0.7%)	104 (77.6%)	2 (1.5%)	22 (14.5%)
	Not Transgender	552 (61.4%)	52 (5.8%)	651 (76.9%)	42 (5.0%)	71 (7.1%)
Sexual Orientation	Gay	251 (79.7%)	47 (14.9%)	239(89.2%)	39 (14.6%)	27 (8.2%)
	Lesbian	129 (51.2%)	0 (0.0%)	168(66.7%)	0 (0.0%)	9 (3.2%)
	Bisexual/ Bicurious	111 (48.5%)	4 (1.7%)	165 (73.7%)	0 (0.0%)	24 (9.4%)
	Pansexual	43 (57.3%)	0 (0.0%)	52 (69.3%)	2 (2.7%)	16 (17.4%)
	Queer	58 (63.7%)	0 (0.0%)	76 (83.5%)	2 (2.2%)	12 (11.9%)
	Identities < 50^b^	26 (42.6%)	2 (3.3%)	45 (76.3%)	1 (1.7%)	3 (4.3%)
Age Bracket	18–25 years old	121 (35.2%)	0 (0.0%)	240 (69.8%)	10 (2.9%)	26 (6.7%)
	26–39 years old	252 (73.5%)	9 (2.6%)	334 (85.3%)	26 (7.8%)	44 (11.7%)
	40–64 years old	218 (77.9%)	34 (12.1%)	245 (78.0%)	7 (2.8%)	22 (7.1%)
	65 + years old	36 (51.4%)	10 (14.3%)	41 (68.3%)	1 (1.7%)	1 (1.3%)
Race	White	505 (63.1%)	36 (4.5%)	610 (79.9%)	30 (3.9%)	56 (6.3%)
	Asian	19 (37.3%)	0 (0.0%)	29 (56.9%)	4 (7.8%)	3 (5.1%)
	Black/African American	22 (52.4%)	42 (7.1%)	29 (74.4%)	1 (2.6%)	7 (14.9%)
	Multiracial	73 (55.7%)	13 (9.9%)	84 (71.2%)	9 (7.6%)	25 (18%)
Ethnicity	Hispanic (Any Race)	130 (60.7%)	22 (10.3%)	129 (67.5%)	9 (4.7%)	40 (17.4%)
Income Bracket^c^	Very Low-Income	116 (54.5%)	9 (4.2%)	139 (68.5%)	3 (1.5%)	37 (15.6%)
	Low-Income	146 (58.6%)	21 (8.4%)	171 (75.0%)	12 (5.3%)	28 (10.3%)
	Mid/High-Income	348 (64.7%)	21 (3.9%)	425 (82.2%)	29 (5.6%)	27 (4.5%)
	Total N (%)	627 (60.5%)	53 (5.1%)	757 (77%)	44 (4.5%)	93 (8.1%)

^a^“Gender Nonconforming” combines nonbinary (8.0%) with gender identity responses with less than 50 respondents including: Gender Fluid 1.8%, Gender Queer 1.8%, Queer 0.5%, Gender Questioning 0.4%, Agender 0.4%, Bigender 0.3%, Androgyne/Androgynous 0.3%, Crossdresser 0.2%, Bisexual 0.2%, Gender Nonconforming 0.2%, Questioning 0.2%. Additional responses, each comprising 0.1% of identities, are: Neither, Asexual, Transgender Nonbinary, Pansexual, Neutrois, Intergender, Transgender, Pangender, Alien

^b^Sexual orientation responses with less than 50 respondents were combined into the category “Identities < 50” including: Asexual 2.6%, Heterosexual 2.2%, Questioning 0.9%. Additional responses, each comprising 0.1% of identities, are: Polysexual, Cupiosexual, Homoromantic Asexual, Aromantic Grey – Asexual

^c^“Very low-income”, “low-income” and “mid/high-income” refers to individuals living in a household earning < 200%, 200–399% and > 400% of the federal poverty level respectively (based on The US Departments of Health & Human Services administrative poverty thresholds defined by household income level and number of persons in the household)

Overall, 8.1% of respondents reported a history of engaging in sex work. Of those individuals who have ever engaged in sex work, 21.7% have never been tested for HIV and 79.6% reported never being offered PrEP for HIV prevention.

### Access to Care

Survey respondents reported many barriers to accessing health care. Overall, 35.5% of survey respondents encountered at least one barrier to care (including cost of care, transportation difficulty, travel time or travel distance) within the prior 12 months (Table 5). Three quarters of respondents (75.0%) have a primary care provider and over half (55.6%) have had a routine preventative care visit within the past year. Nearly half of respondents (46.9%) would prefer to receive primary care from home via telehealth with a LGBTQ + sensitive provider.

**Table 5:** Access to care

Demographic Category	Subcategory	Lack of insurance (at time of survey)	Cost, transportation, travel time OR distance prevented medical care (past year)	Do NOT have a primary care provider	Had a routine check-up within past year	Prefer primary care via telehealth	Nonaffirming experience with provider or office staff	Always or sometimes feel discriminated against in medial settings due to race/ethnicity
Gender Identity	Male	42 (10.9%)	132 (31.4%)	110 (26.1%)	226 (53.8%)	177 (42.2%)	157 (37.3%)	70 (17.6%)
	Female	22 (4.3%)	183 (33.4%)	126 (23.0%)	309 (56.5%)	254 (46.4%)	176 (32.1%)	85 (16.0%)
	Gender Nonconforming^a^	3 (5.0%)	91 (52.6%)	50 (28.9%)	96 (55.5%)	105 (60.7%)	87 (50.3%)	23 (14.8%)
Transgender Status	Transgender	7 (4.7%)	89 (58.9%)	38 (25.2%)	87 (57.6%)	85 (56.3%)	91 (60.3%)	25(17.5%)
	Not Transgender	62 (6.7%)	319 (32%)	249 (25.0%)	550 (55.3%)	453 (45.5%)	334 (33.5%)	155 (16.3%)
Sexual Orientation	Gay	37 (12.5%)	92 (27.9%)	79 (23.9%)	177 (53.6%)	150 (45.7%)	126 (38.2%)	56 (17.9%)
	Lesbian	4 (1.6%)	72 (25.3%)	55 (19.3%)	165 (58.1%)	120 (42.1%)	100 (35.1%)	31 (11.2%)
	Bisexual/ Bicurious	17 (6.7%)	117 (45.5%)	79 (30.7%)	131 (51.0%)	119 (46.3%)	72 (28.0%)	49 (19.9%)
	Pansexual	5 (5.4%)	52 (56.5%)	26 (28.3%)	92 (60.9%)	53 (57.6%)	39 (42.3%)	13 (14.6%)
	Queer	4 (4.0%)	41 (40.6%)	25 (24.8%)	58 (57.4%)	60 (59.4%)	53 (52.4%)	18 (19.6%)
	Identities < 50^b^	1 (1.5%)	29 (42.0%)	18 (26.1%)	42 (60.9%)	29 (42.0%)	27 (39.1%)	10 (15.1%)
Age Bracket	18–25 years old	15 (3.9%)	166 (42.7%)	107 (27.5%)	213 (54.8%)	201 (51.6%)	114 (29.3%)	57 (15.6%)
	26–39 years old	30 (8%)	159 (42.1%)	117 (31.0%)	188 (49.7%)	192 (50.8%)	167 (44.2%)	73 (20.3%)
	40–64 years old	24 (7.8%)	74 (24.0%)	60 (19.5%)	177 (57.8%)	124 (40.4%)	126 (40.9%)	48 (16.0%)
	65 + years old	N/A^c^	9 (12.0%)	3 (4.0%)	60 (80.0%)	22 (29.3%)	18 (24.0%)	2 (2.9%)
Race	White	28 (3.4%)	272 (30.6%)	204 (22.9%)	509 (57.3%)	394 (44.3%)	321 (36.1%)	60 (7.0%)
	Asian	2 (3.4%)	26 (44.8%)	11 (19.0%)	37 (63.8%)	28 (48.2%)	14 (24.1%)	20 (41.7%)
	Black/African American	0 (0.0%)	13 (27.7%)	9 (19.1%)	27 (57.4%)	21 (44.6%)	11 (23.4%)	22 (50.0%)
	Multiracial	37 (27.0%)	87 (62.6%)	56 (40.3%)	59 (42.4%)	89 (64.0%)	73 (52.5%)	75 (59.5%)
Ethnicity	Hispanic (Any Race)	53 (23.6%)	112 (48.5%)	88 (38.1%)	94 (40.7%)	138 (59.7%)	109 (47.2%)	110 (50.7%)
Income Bracket^d^	Very Low-Income	38 (16.2%)	139 (58.2%)	91 (38.1%)	113 (47.3%)	137 (57.3%)	100 (41.9%)	64 (28.5%)
	Low-Income	24 (9.3%)	116 (45.2%)	72 (26.5%)	145 (53.3%)	136 (50.0%)	115 (42.3%)	60 (23.2%)
	Mid/High-Income	6 (1.1%)	138 (23.2%)	117 (19.7%)	350 (59.0%)	247 (41.6%)	199 (33.4%)	50 (8.8%)
	Total N (%)	69 (6.4%)	408 (35.5%)	288 (25.0%)	638 (55.6%)	539 (46.9%)	426 (37.0%)	181 (16.5%)

^a^“Gender Nonconforming” combines nonbinary (8.0%) with gender identity responses with less than 50 respondents including: Gender Fluid 1.8%, Gender Queer 1.8%, Queer 0.5%, Gender Questioning 0.4%, Agender 0.4%, Bigender 0.3%, Androgyne/Androgynous 0.3%, Crossdresser 0.2%, Bisexual 0.2%, Gender Nonconforming 0.2%, Questioning 0.2%. Additional responses, each comprising 0.1% of identities, are: Neither, Asexual, Transgender Nonbinary, Pansexual, Neutrois, Intergender, Transgender, Pangender, Alien

^b^Sexual orientation responses with less than 50 respondents were combined into the category “Identities < 50” including: Asexual 2.6%, Heterosexual 2.2%, Questioning 0.9%. Additional responses, each comprising 0.1% of identities, are: Polysexual, Cupiosexual, Homoromantic Asexual, Aromantic Grey—Asexual

^c^Health insurance coverage was only asked to respondents 18–64 years of age to exclude the Medicare population

^d^“Very low-income”, “low-income” and “mid/high-income” refers to individuals living in a household earning < 200%, 200–399% and > 400% of the federal poverty level respectively (based on The US Departments of Health & Human Services administrative poverty thresholds defined by household income level and number of persons in the household)

Over a third (37.0%) of respondents reported a disrespectful or non-affirming experience by any health care provider or their staff. Of these respondents, negative experiences were reported in all types of care but most frequently in primary care (53.4%). The majority (69.1%) said these experiences made them less likely to seek medical care or otherwise affected the way they seek health care. Additionally, 16.5% of all respondents reported feeling discriminated against in health care settings because of their race or ethnicity.

When adjusted to account for demographic differences, survey participants had higher rates of lack of health insurance (6.5% adjusted) compared to Suffolk (4.5%), though lower rates than NYS (13.6%) and US (8.7%). Cost prevented care for 14.8% of respondents, which was higher than NYS (11.0%) and similar to Suffolk (15.1%) and US (12.9%) rates. Transportation prevented care among 8.1% of respondents, similar to Suffolk (8.9%) and US (8.9%). Travel time/distance interfered with care more among survey participants (19.9% adjusted) compared to Suffolk (15.4%). Survey participants were less likely to have had an annual preventive care visit within the last year (60.8% adjusted) compared to Suffolk (75.3%), NYS (81.0%), and US (70.5%).

### Harassment and Violence

Over two thirds of survey respondents (67.2%) have been verbally harassed because of their SOGI; almost one third (31.7%) physically harassed or attacked because of perceived bias against their SOGI (Table 6). Almost half of respondents (47.1%) reported experiencing unwanted sexual contact; nearly half (45.2%) have experienced emotional intimate partner violence; and over one quarter (27.3%) experienced physical intimate partner violence.

**Table 6:** Harassment and violence

Demographic category	Subcategory	Ever verbally harassed based on SOGI^a^	Ever physically harassed based on SOGI^a^	History of unwanted sexual contact	Ever experienced emotional abuse by intimate partner	Ever experienced physical abuse by intimate partner
Gender Identity	Male	267 (68.4%)	133 (34.1%)	148 (37.9%)	161 (41.3%)	105 (27.0%)
	Female	320 (62.9%)	139 (27.3%)	251 (49.6%)	236 (46.4%)	139 (27.4%)
	Gender Nonconforming^b^	127 (78.4%)	65 (40.1%)	99 (61.1%)	82 (51.0%)	44 (27.3%)
Transgender Status	Transgender	108 (74.5%)	51 (35.2%)	74 (51.1%)	72 (50.0%)	41 (28.3%)
	Not Transgender	607 (66.0%)	287 (31.2%)	425 (46.3%)	409 (44.5%)	248 (27.1%)
Sexual Orientation	Gay	217 (70.4%)	107 (34.8%)	112 (36.4%)	120 (39.0%)	81 (26.5%)
	Lesbian	174 (65.2%)	77 (28.8%)	115 (43.4%)	126 (47.2%)	76 (28.6%)
	Bisexual/ Bicurious	145 (61.4%)	61 (25.9%)	127 (53.8%)	104 (44.1%)	53 (22.5%)
	Pansexual	60 (72.3%)	30 (36.1%)	57 (68.7%)	52 (62.6%)	35 (42.1%)
	Queer	67 (69.8%)	35 (36.5%)	59 (62.1%)	46 (47.9%)	25 (26.0%)
	Identities < 50^c^	43(69.4%)	21 (33.8%)	26 (41.9%)	28 (45.9%)	17 (27.4%)
Age Bracket	18–25 years old	230 (64.4%)	92 (25.7%)	154 (43.3%)	126 (35.4%)	55 (15.4%)
	26–39 years old	257 (72.6%)	124 (35.1%)	206 (58.3%)	195 (55.1%)	122 (34.5%)
	40–64 years old	190 (66.4%)	97 (33.9%)	119 (41.6%)	139 (48.6%)	102 (35.8%)
	65 + years old	40 (57.2%)	14 (35.7%)	22 (31.4%)	22 (31.4%)	11 (15.9%)
Race	White	564 (68.0%)	250 (30.1%)	381 (46.0%)	371 (44.8%)	216 (26.1%)
	Asian	23 (44.2%)	12 (23.0%)	14 (26.9%)	14 (26.9%)	5 (9.6%)
	Black/African American	22 (52.3%)	14 (33.3%)	20 (47.6%)	17 (40.5%)	13 (31.0%)
	Multiracial	100 (77.5%)	57 (44.2%)	80 (62.5%)	76 (58.9%)	55 (42.7%)
Ethnicity	Hispanic (Any Race)	151 (71.3%)	85 (40.1%)	118 (55.9%)	120 (56.6%)	92 (43.6%)
Income Bracket^d^	Very Low-Income	154 (71.7%)	78 (36.3%)	118 (54.9%)	115 (53.4%)	76 (35.5%)
	Low-Income	182 (70.6%)	90 (34.9%)	138 (53.7%)	132 (51.2%)	83 (32.3%)
	Mid/High-Income	357 (64.6%)	158 (28.6%)	230 (41.7%)	224 (40.5%)	124 (22.5%)
	Total N (%)	717 (67.2%)	338 (31.7%)	501 (47.1%)	482 (45.2%)	290 (27.3%)

^a^SOGI is sexual orientation or gender identity

^b^“Gender Nonconforming” combines nonbinary (8.0%) with gender identity responses with less than 50 respondents including: Gender Fluid 1.8%, Gender Queer 1.8%, Queer 0.5%, Gender Questioning 0.4%, Agender 0.4%, Bigender 0.3%, Androgyne/Androgynous 0.3%, Crossdresser 0.2%, Bisexual 0.2%, Gender Nonconforming 0.2%, Questioning 0.2%. Additional responses, each comprising 0.1% of identities, are: Neither, Asexual, Transgender Nonbinary, Pansexual, Neutrois, Intergender, Transgender, Pangender, Alien

^c^Sexual orientation responses with less than 50 respondents were combined into the category “Identities < 50” including: Asexual 2.6%, Heterosexual 2.2%, Questioning 0.9%. Additional responses, each comprising 0.1% of identities, are: Polysexual, Cupiosexual, Homoromantic Asexual, Aromantic Grey – Asexual

^d^“Very low-income”, “low-income” and “mid/high-income” refers to individuals living in a household earning < 200%, 200–399% and > 400% of the federal poverty level respectively (based on The US Departments of Health & Human Services administrative poverty thresholds defined by household income level and number of persons in the household)

## Discussion

This is the first comprehensive health needs assessment of LGBTQ+ adults residing or enrolled in an educational program in Nassau/Suffolk Counties in NYS. Results underscore health disparities for this community, which are largely consistent with national and international findings (Cochran et al., 2003; European Union Agency for Fundamental Rights, 2013; Institute of Medicine, 2011; James et al., 2016). Many respondents indicated profound mental health concerns including > 60% reporting symptoms of chronic depression, almost half with fair/poor mental health and more than a third with thoughts of self-harm. Results also revealed high levels of smoking, excessive drinking, and illicit drug use. Notably, all direct measures of mental health and most, if not all, measures of substance use are unfavorable relative to rates in Suffolk and the US. Higher rates of mental health issues and substance use among the LGBTQ+ population has been attributed to *minority stress theory*, whereby experienced or anticipated stigma and prejudice create a stressful environment causing internalizing homophobia/transphobia, depression, anxiety and concealment of identity (Ard & Makadon, 2016; Dentato, 2012; Meyer, 2003).

Despite such high rates of mental health concerns, just over one-third reported receiving mental health treatment. Respondents were more likely to be receiving mental health treatment compared to the general US population, which is consistent with prior studies (Cochran et al., 2003; Platt et al., 2018). Mental health treatment may be more accepted and less stigmatized among the LGBTQ + community; however, there is still a tremendous unmet need for services. This remains a high priority area that needs to be addressed.

In this study, more than a third of respondents have ever experienced disrespectful or non-affirming treatment in health care, which is also consistent with prior studies (Bradford et al., 2013; European Union Agency for Fundamental Rights, 2013; Jaffee et al., 2016; James et al., 2016; Medina et al., 2021; Shires & Jaffee, 2015). These experiences were reported across a wide array of health care settings. For most, these experiences impacted their subsequent health care usage, which is consistent with prior studies (Jaffee et al., 2016; James et al., 2016). It is imperative that providers receive cultural sensitivity training to understand the disparities that exist among LGBTQ + individuals; the negative effects of heteronormative assumptions; and the positive downstream effects of providing affirming, respectful care, where patients feel welcomed to disclose their identity. It may also be beneficial to create a directory of LGBTQ + friendly providers who are knowledgeable about pertinent health issues and have experience providing affirming care.

While results are largely consistent with national findings (Cochran et al., 2003; Institute of Medicine, 2011; James et al., 2016), the survey data also uncovered disparities between the SOGI subgroups. Participants self-reported > 20 different sexual orientations and > 30 gender identities, underscoring the diverse ways by which people identify their sexual orientation and gender. People who identified as bisexual/bicurious, pansexual, or queer (bi+); and gender non-conforming or transgender reported more mental health and health care access difficulties than people who identified as male, female, gay man, or lesbian. These results may be partially explained by unique challenges that gender nonconforming individuals encounter like being ignored, misgendered and excluded from the binary gender community. Bi+ individuals face similar challenges with misperceptions about sexual orientation and being excluded from the gay/lesbian community (Fiani & Han, 2018; Matsuno & Budge, 2017). Consequently, some of these subpopulations may not receive necessary and appropriate health care services, or may conceal their identity due to fear of discrimination, all of which are associated with negative health outcomes (Feinstein et al., 2020; Ferlatte et al., 2020; Schrimshaw et al., 2013). As such, it is important that health care organizations work closely with community partners to outreach and engage members of these subgroups to assist with accessing affirming and culturally sensitive care and other resources.

Results also varied by other demographics like age range. Young adults aged 18–26 experienced high rates of mental difficulties and barriers to care, with lower rates of preventive health services and HIV testing. Participants aged 26–39 experienced remarkably high rates of substance use and intimate partner violence compared to the overall sample. Research suggests that an LGBTQ + individual’s stressors progress from proximal or internalizing stressors (like internal stigma, concealment, fear of disclosure and rejection) during adolescence to more distal stressors including interpersonal stressors (like family rejection or discrimination in the workplace) and structural level stressors (like discriminatory laws and political climate). LGBTQ + young adults are more likely to use negative or avoidant coping mechanisms like substance use to help manage these stressors (Felner et al., 2020; Krueger et al., 2021). Additionally, sociocultural norms and media portrayal stress substance use as a key aspect of young adult LGBTQ + socialization (Demant et al., 2021; Felner et al., 2020).

Regarding race and ethnicity, Asian individuals reported very high rates of mental health difficulties and lower than average rates of mental health treatment, which is consistent with prior studies (Lee et al., 2009; Nishi, 2012; Spencer et al., 2010). Factors that may contribute to these trends include parental and familial pressures to succeed, mental health being a taboo topic in many Asian cultures and the need to uphold the “Model Minority” stereotype. Black, multiracial and Hispanic respondents reported increased rates of substance use, mental health difficulties, barriers to care, and low utilization of preventive care compared to the overall sample. The intersection of minority stress due to SOGI and discrimination stemming from race and ethnicity may partially explain these results (Ard & Makadon, 2016; Institute of Medicine, 2011). Additionally, LGBTQ+ people of color may be more likely to experience discrimination and rejection from the LGBTQ+ community.

Socioeconomic status also significantly impacted results. Individuals categorized as low-income experienced higher rates of all mental health difficulties and increased rates of tobacco and drug use compared to mid/high-income individuals; and very low-income individuals experienced even higher rates. Furthermore, low-income individuals experienced higher barriers to care and lower rates of preventive services, including an annual check up and knowledge or use of HIV PrEP. LGBTQ+ individuals are more likely to experience poverty and economic hardship (James et al., 2016). Underlying factors contributing to this disparity may include high dropout rates due to discrimination or harassment in school and unemployment due to discrimination when seeking employment (James et al., 2016).

A key component of a community health needs assessment involves communicating the survey results internally (to the collaborative partners) and externally (to the science and lay communities). We shared results through oral presentations to community partners and professional conferences, a press release event (including online, broadcast and print media), hospital newsletter, and created a survey microsite (available in English and Spanish) highlighting key findings with infographics. It is essential that results drive an action plan to help address the disparities identified in the assessment. We have implemented improvements and are working towards a comprehensive action plan targeting expanding clinical services, increasing behavioral health services (e.g., support groups), public health initiatives, expanding training for medical providers and staff as well as community organizations, adding LGBTQ+ material into health science education curricula, and increasing community engagement.

### Limitations

This study utilized snowball sampling. While this method may aid in the recruitment of “hidden” participants more effectively, it does introduce sampling bias and participants may not necessarily represent the true distribution of LGBTQ+ adults. The survey sample size is sizeable which may help reduce selection bias. Although steps were taken to increase participation from underrepresented communities including non-English speakers and those with limited literacy, these groups may not have been fully represented in the sample. Comparisons should be interpreted with some caution given this survey was conducted during the COVID-19 pandemic, whereas the comparative benchmark data were collected prior to the pandemic.

## Conclusions/Policy Implications

Overall, findings provide important insight into the foci that should be addressed in efforts to improve LGBTQ+ health and reduce disparities. First, in order to make health care more welcoming for all members of the community, a more culturally sensitive and informed health care workforce must be prioritized. Implementing cultural sensitivity training for providers and staff which includes bias-focused education and experiential learning interventions can increase knowledge and comfort levels (Morris et al., 2019). LGBTQ+ health should also be incorporated into health care students' curricula. Second, given the extreme high rates of mental health difficulties, there is a clear need for additional mental health resources to support Nassau/Suffolk Counties' LGBTQ+ community. Innovative strategies like the use of telehealth services, online virtual support groups and integrating behavioral health into primary care practices may improve access to mental health services (Heredia et al., 2021; Whaibeh et al., 2020). Last, this survey intentionally focused on identifying problems or deficits as a means of galvanizing action. However, research has identified resilience factors, like social support, that promotes wellbeing and a sense of connectedness among LGBTQ + individuals (Heredia et al., 2021; Hudson & Romanelli, 2020). Throughout this study, community partnerships were essential to connect with the LGBTQ+ population. Community acceptance and support is strongly associated with overall wellness (Hudson & Romanelli, 2020) and may help counteract health disparities. As such, collaboration with community partners to create inclusive events and resources may help build supportive communities that reinforce belonging and resilience.

## Data Availability

The datasets generated and/or analyzed during the current study are not publicly available but are available from the corresponding author on reasonable request.
